# Clinical Implications of Chloroquine and Hydroxychloroquine Ototoxicity for COVID-19 Treatment: A Mini-Review

**DOI:** 10.3389/fpubh.2020.00252

**Published:** 2020-05-29

**Authors:** Pattarawadee Prayuenyong, Anand V. Kasbekar, David M. Baguley

**Affiliations:** ^1^Hearing Sciences, Division of Clinical Neuroscience, School of Medicine, University of Nottingham, Nottingham, United Kingdom; ^2^NIHR Nottingham Biomedical Research Centre, University of Nottingham, Nottingham, United Kingdom; ^3^Department of Otorhinolaryngology, Nottingham University Hospitals NHS Trust, Nottingham, United Kingdom; ^4^Department of Otorhinolaryngology Head and Neck Surgery, Faculty of Medicine, Prince of Songkla University, Songkhla, Thailand

**Keywords:** chloroquine, hydroxychloroquine, ototoxicity, hearing loss, tinnitus, COVID-19

## Abstract

At this time of the COVID-19 pandemic, potentially effective treatments are currently under urgent investigation. Benefits of chloroquine and hydroxychloroquine for the treatment of COVID-19 infection have been proposed and clinical trials are underway. Chloroquine and hydroxychloroquine, typically used for the treatment of malaria and autoimmune diseases, have been considered for off-label use in several countries. In the literature, there are reports of ototoxic effects of the drugs causing damage to the inner ear structures, which then result in hearing loss, tinnitus, and/or imbalance. This mini-review represents a summary of the findings from a systematic search regarding ototoxicity of chloroquine and hydroxychloroquine in the published literature. The characteristics of sensorineural hearing loss and/or tinnitus after chloroquine or hydroxychloroquine treatment can be temporary but reports of persistent auditory and vestibular dysfunction exist. These are not frequent, but the impact can be substantial. Additionally, abnormal cochleovestibular development in the newborn was also reported after chloroquine treatment in pregnant women. The suggested dose of chloroquine for COVID-19 infection is considerably higher than the usual dosage for malaria treatment; therefore, it is plausible that the ototoxic effects will be greater. There are potential implications from this review for survivors of COVID-19 treated with chloroquine or hydroxychloroquine. Patient reports of hearing loss, tinnitus, or imbalance should be noted. Those with troublesome hearing loss, tinnitus and/or imbalance are encouraged to be referred for hearing evaluation and interventions once they are stable. Clinical trials of chloroquine or hydroxychloroquine should also consider including audiological monitoring in the protocol.

## Introduction

At this time of the COVID-19 global pandemic, potentially effective treatments are currently under urgent investigation. Currently, there is no evidence from randomized clinical trials that any specific therapy improves outcomes in patients with COVID-19 (1). Chloroquine and hydroxychloroquine are considered to be promising repurposed drugs against COVID-19, based on pathophysiological considerations and *in vitro* results (2, 3). These drugs have received particular attention as they are widely available and inexpensive. Chloroquine and hydroxychloroquine, quinine-related compounds, have been used for the treatment of malaria and chronic inflammatory diseases such as systemic lupus erythematosus and rheumatoid arthritis. The anti-viral and anti-inflammatory properties may account for the efficacy in treating patients with COVID-19 infection (4). There have been reports that patients who received chloroquine or hydroxychloroquine had faster virological clearance (5, 6), however there are some limitations of the studies such as small sample size and questionable methodology. There is no high-quality evidence of potential benefit of these drugs at the moment. Presently, there are over 80 registered ongoing trials worldwide examining the role of chloroquine and hydroxychloroquine in COVID-19 treatment (7).

Clinical practice guidelines have considered chloroquine and hydroxychloroquine for off-label and compassionate therapies against moderate to severe cases of COVID-19 in several countries including China, Korea, USA, France, Italy, and Belgium (8). There is currently also a massive global demand for chloroquine and hydroxychloroquine as people around the world are self-medicating after health professionals and politicians have endorsed the drugs. Chloroquine and hydroxychloroquine are also freely available in the UK and other countries without prescription.

Some potential side effects of chloroquine and hydroxychloroquine are cardiac arrhythmias, retinopathy, and muscle weakness (4). The clinical and research literature also contains reports of ototoxic effects after chloroquine and hydroxychloroquine treatment. Ototoxicity refers to drug-related injury causing damage to the inner ear structures, which then result in hearing loss and/or tinnitus (the subjective perception of sound such as ringing, hissing, or buzzing, without an external source), and/or imbalance (9). Permanent hearing loss can adversely affect cognitive health (10) and mental well-being (11). Troublesome tinnitus is associated with insomnia, poor concentration, anxiety and depression (12). The mechanisms of chloroquine associated hearing loss include cochlear outer hair cell dysfunction, and inhibition of post synaptic sodium channel function in cochlear spiral ganglion cells (13). Additionally, some alterations in central auditory function, which may trigger tinnitus, have been observed after quinine administration (13).

This mini-review represents a summary of the findings from a literature search regarding ototoxicity of the drugs in the published literature as well as the discussion of potential implications for survivors of COVID-19 so treated.

## Method

A systematic literature search on Medline and EMBASE platforms was undertaken on 26th March 2020, updated on 23rd April 2020. The search strategy combined MeSH terms and keywords of chloroquine or hydroxychloroquine, ototoxicity, hearing loss, hearing, tinnitus. English language publications containing relevant data to this review were included. Data extraction items included year, study design, sample size, and audiological outcomes. Data were collated in the table and then summarized by narrative synthesis. Recommendations from audiological professional perspectives were then made.

## Results

### Chloroquine Ototoxicity

Eleven publications, reporting topics associated with ototoxic effects of chloroquine, were identified and are summarized in Table 1. The year of publication ranged from 1954 to 2015. There were 7 case reports, 2 observational studies, 1 case control study, and 1 review article. The sample size of the study participants varied from 1 to 74.

**Table 1:** Chloroquine ototoxicity.

**Title**	**References**	**Study type**	**Sample size**	**Dosage**	**Results**
Assessment of short term chloroquine-induced ototoxicity in malaria patients (14)	Subramaniam and Vaswani (14)	Prospective observational study	30 (Aged 14-58 years old)	1200 mg loading load then 600 mg oral every 12 hours for 2 days	- 2 subjects showed a change in hearing thresholds on high frequency audiometry (8-12 kHz). Pure tone audiometry showed bilateral mild sensorineural hearing loss at 12 kHz in 1 subject, and bilateral mild to moderate sensorineural hearing loss at 8 kHz in another. The otoacoustic emission (OAE) and auditory brainstem response (ABR) findings were also abnormal in these 2 subjects. A 1 month follow-up Pure Tone Audiogram was normal. - 1 subject showed vestibular side effects in the form of ‘giddiness' and nystagmus which spontaneously resolved on completion of therapy.
Chloroquine ototoxicity (15)	Bortoli and Santiago M (15)	Review	–	–	– Some reports have described sensorineural hearing loss, tinnitus, sense of imbalance after prolonged high dose of chloroquine. - The reversibility of chloroquine ototoxicity has been debatable.
Chloroquine gestational use in systemic lupus erythematosus: assessing the risk of child ototoxicity by pure tone audiometry (16)	Borba et al. (16)	Case-control study	19 (Mean age of mothers was 27 years old)	250 mg daily	- There was no difference in hearing thresholds by pure tone audiometry of children between chloroquine exposure and non-exposure groups during the gestation. The mean hearing thresholds (Pure Tone Audiometry) at low frequencies of exposure and non-exposure groups were similar (11.4 ± 4.5 vs. 11.9 ± 3.0 dB; *p* = 0.66). The mean hearing threshold at high frequencies of exposure and non-exposure groups were not significantly different (8.5 ± 5.0 vs. 7.4 ± 3.6 dB; *p* = 0.55).
Chloroquine ototoxicity: an idiosyncratic phenomenon (17)	Hadi et al. (17)	Case report of a 2.5-year-old boy	1	No information	- Abnormal gait a few hours after single chloroquine intramuscular injection. Severe hearing loss on the 2nd day. 10 days later, he was treated with steroid and plasma expander. He still had permanent severe hearing loss at 3-5 years follow up. No testing technique information given.
Alterations of auditory evoked potentials during the course of chloroquine treatment (18)	Bernard (18)	Observational study	74	No information	- There was no hearing change by pure tone audiogram. - There were abnormal results of brainstem audiometry (Auditory Brainstem Audiometry) in 13 patients which resolved after chloroquine discontinuation.
Chloroquine ototoxicity—a reversible phenomenon? (19)	Mukherjee (19)	Case report of a 6-year-old girl	1	250 mg intramuscular injection daily for 7 days	- She complained of hearing loss, and had abnormal gait after chloroquine injection for malaria. Pure Tone Audiometry indicated severe unilateral sensorineural hearing loss, which was worse at mid frequencies (mean hearing threshold at mid frequencies = 80 dB) - Patient hearing improved after prednisolone administration, and Pure Tone Audiometry indicated the mean hearing threshold at mid frequencies was 35 dB bilaterally.
Ototoxicity of chloroquine phosphate: a case report (20)	Dwivedi and Mehra (20)	Case report of a 52-year-old man	1	1,000 mg loading load	- The patient had bilateral permanent deafness, severe vomiting, vertigo, blurring of vision and tinnitus at 1.5 hour after taking a single dose of 1g of chloroquine. Pure tone audiometry indicated hearing thresholds of more than 90 dB in both ears.
Sudden deafness and chloroquine injection: personal communication	Obiako (personal communication)	Case report	4	No information	- There were 4 cases of sensorineural deafness following chloroquine phosphate injections. No testing technique information given.
Ototoxicity of chloroquine (21)	Matz and Naunton (21)	Case report	1	No information	- There was a complete absence of inner and outer hair cells throughout the length of the cochlea in a deaf child whose mother took chloroquine during pregnancy. No testing technique information given.
The ototoxicity of chloroquine phosphate (22)	Hart and Naunton (22)	Case report	2	No information	- There were 2 cases of severe bilateral cochleovestibular paresis whose mothers were treated with chloroquine during her pregnancy.No testing technique information given.
Chloroquine in lupus erythematosus	Dewar and Mann (23)	Case report	1	No information	- The patient had irreversible sensorineural deafness, after being treated with chloroquine for 7 months. No testing technique information given.

Ten patients (8 adults and 2 children) in 6 publications had either abnormal audiogram or reported hearing loss after chloroquine treatment. Three out of ten cases had temporary sensorineural hearing loss after chloroquine treatment that improved after cessation of the medication (14, 19). A prospective observational study in 2015 concluded that ototoxic effects of chloroquine at regular doses for malaria treatment (1.2 g daily for 3 days) were fully reversible (14). Sensorineural hearing loss after chloroquine in a 6-year old girl was partially reversible after prednisolone administration (19). However, permanent severe sensorineural hearing loss has also been reported in 2 cases (17, 20). Additionally, reversible chloroquine-induced cochlear injury was detectable by brainstem audiometry in 13 out of 70 patients despite normal pure tone audiogram results (18). Tinnitus has also been reported concurrently with persistent hearing loss in 1 case (20). Imbalance was reported in 3 cases (14, 19, 20).

While there was no difference in hearing thresholds between children who were and were not exposed to chloroquine during gestation (16), there were 3 case reports of intrauterine effects of chloroquine associated with abnormal cochleovestibular development in newborns (21, 22).

### Hydroxychloroquine Ototoxicity

Six case reports, describing ototoxic effects associated with hydroxychloroquine, were identified and are displayed in Table 2. Publication year ranged from 1998 to 2018. Sensorineural hearing loss was identified after hydroxychloroquine treatment in five adults and two children. The sensorineural hearing loss was found to be either reversible (25, 28) or irreversible (24, 29). The onset of hearing loss after hydroxychloroquine treatment varied from 1 month (25) to several years (29). Tinnitus was also reported concomitantly with hearing loss in 2 cases (24, 28).

**Table 2:** Hydroxychloroquine ototoxicity.

**Title**	**References**	**Study type**	**Sample size**	**Dosage**	**Results**
Hydroxychloroquine ototoxicity in a patient with systemic lupus erythematosus (24)	Fernandes et al. (24)	Case report of a 51-year-old woman	1	400 mg daily	- Patient complained of tinnitus and bilateral hearing loss, 3 years after sustained hydroxychloroquine use. Pure Tone audiometry indicated moderate sensorineural hearing loss in the left ear, and mild to moderate in the right ear. Her hearing loss and tinnitus persisted after the discontinuation of medication.
A case report of hearing loss post use of hydroxychloroquine in a HIV-infected patient (25)	Khalili et al. (25)	Case report of a 57-year old man	1	400 mg daily	- Bilateral slowly progressive reversible sensorineural hearing loss after 1 month of hydroxychloroquine. Pure Tone Audiometry indicated moderate to severe sensorineural hearing loss bilaterally. Two months after the discontinuation of hydroxychloroquine, audiometric findings improved, showing mild to moderate hearing loss in both ears.
Hydroxychloroquine-induced ototoxicity in a child with systemic lupus erythematosus (26)	Lim and Tang (26)	Case report of a 11-year old girl	1	100 mg daily (3 mg/kg/d)	- Patient complained of reduced hearing after 2 months of hydroxychloroquine use. Pure Tone Audiometry indicated bilateral sensorineural hearing loss, predominantly affecting the low-frequency range.
Hydroxychloroquine ototoxicity in a child with idiopathic pulmonary haemosiderosis (27)	Coutinho and Duarte (27)	Case report of a 7-year-old girl	1	200 mg daily	- Patient had unilateral slowly progressive hearing loss after 2 years of hydroxychloroquine use. Pure Tone Audiometry indicated moderate to severe sensorineural hearing loss (mean hearing threshold 65 dB). The auditory brainstem response (ABR) test showed absence of response at 90 dB in the right ear.
Hydroxychloroquine ototoxicity in a patient with rheumatoid arthritis (28)	Seckin et al. (28)	Case report of a 34-year-old woman	1	400 mg daily	- Patient complained of hearing loss and tinnitus after 5 months of hydroxychloroquine use. Pure Tone Audiometry indicated bilateral mild sensorineural hearing loss. After discontinuation of hydroxychloroquine, patient symptoms improved and the follow-up audiogram was normal.
Otoxicity due to hydroxychloroquine: report of two cases (29)	Johansen and Gran (29)	Case report of a 44-year-old woman and a 44-year-old man	2	No information	- Patients had irreversible sensorineural hearing loss after several years of hydroxychloroquine use. No testing technique information given.

### Discussion

The manifestation of sensorineural hearing loss and/or tinnitus and/or imbalance after chloroquine and hydroxychloroquine can be either temporary or permanent. Most of the studies on this topic were case series or case reports with only a few observational studies. Information from a definitive large study with good methodology is still lacking. Ototoxicity after chloroquine use tends to be more sudden, while hydroxychloroquine is more likely to cause ototoxicity after prolonged use. This could be due to different drug efficacy and equivalent dosage. Furthermore, hearing loss in these patients could be associated with other possible causes rather than chloroquine and hydroxychloroquine including autoimmune disease e.g., systemic lupus erythematosus (30), sudden sensorineural hearing loss or presbycusis.

The suggested dose of chloroquine for patients diagnosed with COVID-19 infection (1 g daily for 10 days) is substantially higher compared with the usual dosage of chloroquine for malaria treatment (1 g daily for 3 days) (5). There is no information regarding the ototoxic effect of chloroquine at this higher dose. Patients with chronic inflammatory diseases were treated with a usual dose of hydroxychloroquine 400 mg daily for long durations (months or years). A suggested dose of hydroxychloroquine for COVID-19 infection is an initial loading dose of 800 mg followed by 400 mg daily for 4 days based on the *in vitro* model (2), and 600 mg daily for 10 days from a French study (6). In general, the recommended dosage of hydroxychloroquine in COVID-19 patients is slightly higher but in a shorter duration compared to that in autoimmune disease. The ototoxic effects of these regimens are unknown.

Due to the potentially substantial number of the world's population who may take chloroquine or hydroxychloroquine, there is the prospect of a significant number of people being affected with ototoxic side effects. It is therefore vital to build awareness about the presentation and impact of the symptoms of drug-induced ototoxicity. Patient reports of hearing loss, tinnitus, or imbalance should be noted. Those with troublesome hearing loss or tinnitus are encouraged to be referred for hearing evaluation, including extended high frequencies audiometry at 8–16 kHz where possible, once they are stable. Available options of audiological interventions for those with bothersome hearing impairment or tinnitus are counseling, hearing aids, and tinnitus therapy. The possibility of exacerbation of pre-existing hearing loss and/or tinnitus should be considered. Synergistic adverse auditory effects when other ototoxic medication is administered with chloroquine or hydroxychloroquine, such as aminoglycoside antibiotics and azithromycin, is a further risk (9). Severe cases of COVID-19 can also progress to respiratory distress and hypoxia (31). Hypoxia is known to have deleterious effects on the stria vascularis of the cochlea organ including alterations to cochlear potentials and histologic changes (32). Therefore, it is certainly possible that the combined effects of hypoxia and administration of chloroquine or hydroxychloroquine on hearing could be worse than either one alone. Clinical trials of chloroquine or hydroxychloroquine should also consider including audiological monitoring in the protocol. Ideally, a hearing test should be conducted both before and after drug administration to examine drug-induced hearing change. Common methods for audiological evaluation include pure tone audiometry, otoacoustic emission (OAE), and tinnitus questionnaire. However, conventional methods and setting of hearing evaluation is impractical based on the infectious nature of COVID-19 and the urgency of drug administration. Self-monitoring by validated smartphone-based apps for hearing assessments in addition to self-report of symptoms is an approach of interest in this situation.

Although chloroquine and hydroxychloroquine are generally considered safe in pregnant women, the use of chloroquine during pregnancy in the first trimester should be contemplated with particular caution since there are reports of abnormal cochleovestibular development in newborns. Hydroxychloroquine has a safer clinical profile in pregnancy, thus is a more suitable option than chloroquine (33).

### Conclusion

Recent publications have brought attention to the possible benefit of chloroquine and hydroxychloroquine in COVID-19 treatment. It is important to build awareness about the possibility of ototoxicity in survivors of COVID-19 treated with these drugs. Patient reports of hearing loss, tinnitus, or imbalance should be noted. Those with troublesome hearing loss or tinnitus are encouraged to be referred for hearing evaluation and interventions once they are stable. Clinical trials of chloroquine or hydroxychloroquine should also consider including audiological monitoring in the protocol.

## Author Contributions

All authors planned and structured the paper. PP undertook the review and wrote the first draft. DB, PP, and AK jointly revised the manuscript.

## Conflict of Interest

The authors declare that the research was conducted in the absence of any commercial or financial relationships that could be construed as a potential conflict of interest.
